# Predicting the Habitat Suitability for Endangered Tree Species *Pterocarpus marsupium* in Nepal, Using Ensemble Species Distribution Models

**DOI:** 10.1002/ece3.73990

**Published:** 2026-07-10

**Authors:** Ripu Kunwar, Satyam Kumar Chaudhari, Shreehari Bhattarai, Binaya Adhikari, Babita Khadka, Gokarna J. Thapa, Anant Bhandari, Man Dev Bhatt

**Affiliations:** ^1^ Gandaki University Pokhara Nepal; ^2^ Central Department of Environmental Science Tribhuvan University Kathmandu Nepal; ^3^ Environment Conservation Forum Jhapa Nepal; ^4^ Agriculture and Forestry University Chitwan Nepal; ^5^ Institute of Forestry Tribhuvan University Kathmandu Nepal; ^6^ Natural Science Society Kathmandu Nepal; ^7^ Siddhanath Science Campus Tribhuvan University Kathmandu Nepal; ^8^ WWF Nepal Kathmandu Nepal

**Keywords:** biomod2, climate change, community forestry, conservation planning, human footprint, *Pterocarpus marsupium*

## Abstract

*Pterocarpus marsupium* Roxb., locally known as Bijaysal, is a high‐value tropical deciduous tree with a restricted and fragmented distribution in Nepal. We integrated 129 spatially occurring records with climatic, anthropogenic, edaphic, and topographic variables in an ensemble species distribution modeling framework (biomod2) to project current and future (2050s and 2090s) habitat suitability under four shared socioeconomic pathways (SSP) scenarios. Minimum temperature of the coldest month and precipitation seasonality were the dominant climatic drivers, followed by soil organic carbon, bulk density, and human footprint. The current suitable habitat is limited (11,154 km^2^; 7.6% of Nepal) and confined to the western and central lowlands. Future projections conditional on the BCC‐CSM2‐MR GCM climatic model indicate potential habitat expansion ranging from ~40% (SSP1‐2.62090s) to 313% (SSP2‐4.52050s) with consistent southeastward centroid shifts, reflecting a leading‐edge expansion into the northern mid‐hills alongside a major redistribution of suitable core habitats toward eastern and southeastern lowlands. However, these projections should be interpreted with caution, as actual migration may be limited by seed dispersal mechanisms, biotic interaction, future land use change, and soil suitability in high‐elevation zones. The human footprint response suggests that conservation strategies outside strictly protected areas may also play an important role. These findings may help identify potential areas for further field assessment, but assisted colonization would require site‐specific soil, dispersal, and genetic feasibility studies before implementation.

## Graphical abstract

Habitat suitability of *Pterocarpus marsupium* across Nepal for 2050 and 2090 under four distinct climate change scenarios, ranging from low to high emission pathways.

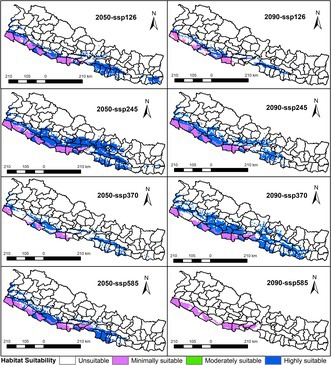

## 1 Introduction

*Pterocarpus marsupium* Roxb. (Fabaceae), commonly known as Bijaysal in Nepali and the Kino tree in English, is a medium to large‐sized deciduous tree that can reach heights of up to 33 m (Duthie 1915; Barstow 2017). It has a limited global and national distribution and occupies a relatively narrow elevational range (Koirala and Pyakurel 2015; GoN 2021). The limited range of distribution, along with its slow growth and poor regeneration (up to 30%), coupled with its overexploitation, poses a serious threat to its extant population and distribution (Koirala and Pyakurel 2015). It has been listed as one of the highly exploited species in western Nepal (Pant and Yadav 2013). *Pterocarpus marsupium* has been placed in the IUCN Red List of threatened species under the status of ‘Near Threatened’ due to its global population reduction (Barstow 2017). It is also listed as Critically Endangered for Nepal under the conservation assessment and management plan (CAMP) threat category (CAMP 2001). In Nepal, *P. marsupium* was declared and listed as protected in 2001, with restrictions on felling, transport, and export of the plant in the country (*Government of Nepal* 1993, 1995, 2001).

Global distribution of *P. marsupium* is restricted as it stretches across the Indian Peninsula up to the north at the lowland of the central Himalayas (Troup 1921). *P. marsupium* is found in tropical South Asia, including India, Nepal, Bangladesh, and Sri Lanka, extending to the lowlands of the central Himalayas at the northernmost distribution limit (Paneru et al. 2021). Primarily distributed in lowland tropical deciduous forest within 100–1200 m altitude range, it is mostly found limited to tropical deciduous forest of Terai and Siwalik in the central and western parts of Nepal and occur as small number, scattered and isolated population in the altitude ranging from roughly 100–640 m elevation in Nepal (DoF 2018; Paneru et al. 2018; Chalise and Khatri 2021). It is found in flat and irregular terrains and on a wide variety of soil types (DoF 2018). It commonly occurs in Sal ( *Shorea robusta* ) dominated mixed deciduous forests associated with species such as Karma (*Adina cordifolia*), Sindure ( *Mallotus philippensis* ), Harro ( *Terminalia chebula* ), *Barro* ( *Terminalia bellirica* ), and Khair ( *Acacia catechu* ) (Troup 1921; Pyakurel and Oli 2014). It has been reported from community, collaborative, and government‐managed forests, as well as from private agricultural lands (DoF 2018; Khanal and Bhattarai 2020).

Although *Pterocarpus marsupium* holds ethnobotanical and economic value for its high‐quality timber, traditional medicinal properties, and fodder (Mishra 1993; Oommen et al. 2000; Tiwari et al. 2004), these multiple‐use values have driven intensive overexploitation and severe anthropogenic pressures across its habitat range (Khanal and Bhattarai 2020). Consequently, the species exhibits profound ecological vulnerability, currently existing in small, fragmented areas and isolated populations restricted to narrow bioclimatic zones in Nepal (DoF 2018). This high level of habitat fragmentation, with specialized niche requirements, increases its long‐term climate sensitivity and risk of localized extinction, making its status as a top conservation priority (Chalise and Dhakal 2021; Kunwar 2022). Natural regeneration of *P. marsupium* is restricted by low seed viability, hard seed coats, slow growth rates, and unfavorable microhabitat conditions (Kalimuthu and Lakshmanan 1995; Koirala and Pyakurel 2015). In addition to these biological constraints, anthropogenic pressures such as habitat destruction and fragmentation, degradation of forests, over‐exploitation, overgrazing, forest fires, construction, etc. All these factors contributed to a sustained decrease in the population and habitat of *P. marsupium* (Pant and Yadav 2013; Kunwar 2022). As a result, low density, which doesn't form contiguous stands, has poor natural regeneration due to a narrow ecological niche. Habitat destruction and degradation have proved to be the primary cause of this continuous decline of the species. Agricultural land has been increasingly substituting forests due to the increasing human population, with the construction of roads and other infrastructure (Pant and Yadav 2013). Further destruction of the existing habitat and suppression of natural regeneration occurred due to various causes, including free grazing, frequent forest fires, and natural disasters (Kunwar 2022). The combined effects of slow growth, poor natural regeneration, restricted distribution, and intensive exploitation for timber, fodder, and furniture have posed serious threats to its extant populations (Pant and Yadav 2013).

Ensemble modeling is widely regarded as one of the most robust approaches for predicting species distributions under climate change scenarios (Araujo and New 2007; Araújo et al. 2005) by integrating multiple factors, including bioclimatic, physiographic, anthropogenic, and edaphic factors (Wiens et al. 2010). It is often advantageous to merge different models to improve prediction accuracy, as this consistently performs better than individual models (Grenouillet et al. 2011). Ensemble modeling prediction produces better results under current and future climate conditions (Latif et al. 2013; Ahmad et al. 2020). It ensures effective conservation and management of biodiversity through appropriate strategies and measures under diverse environmental conditions (Dawson et al. 2011).

In this context, we simulated an ensemble of species distribution models to assess the spatial–temporal distribution pattern of *Pterocarpus marsupium* under climate change scenarios, as well as identify the underlying driving factors. We hypothesized that: (a) future climate warming could trigger a range expansion or upward elevational shift into the mid‐hills if dispersal and establishment constraints are overcome, and (b) winter temperature limits and moisture availability will act as primary climatic drivers of its distribution, while localized suitability will be heavily dictated by edaphic properties and intermediate levels of human disturbance. Furthermore, we discuss the conservation implications for *P. marsupium* by identifying key future climate refugia and exploring adaptive management strategies.

## 2 Materials and Methods

### 2.1 Species Occurrence Records

**FIGURE 1:**
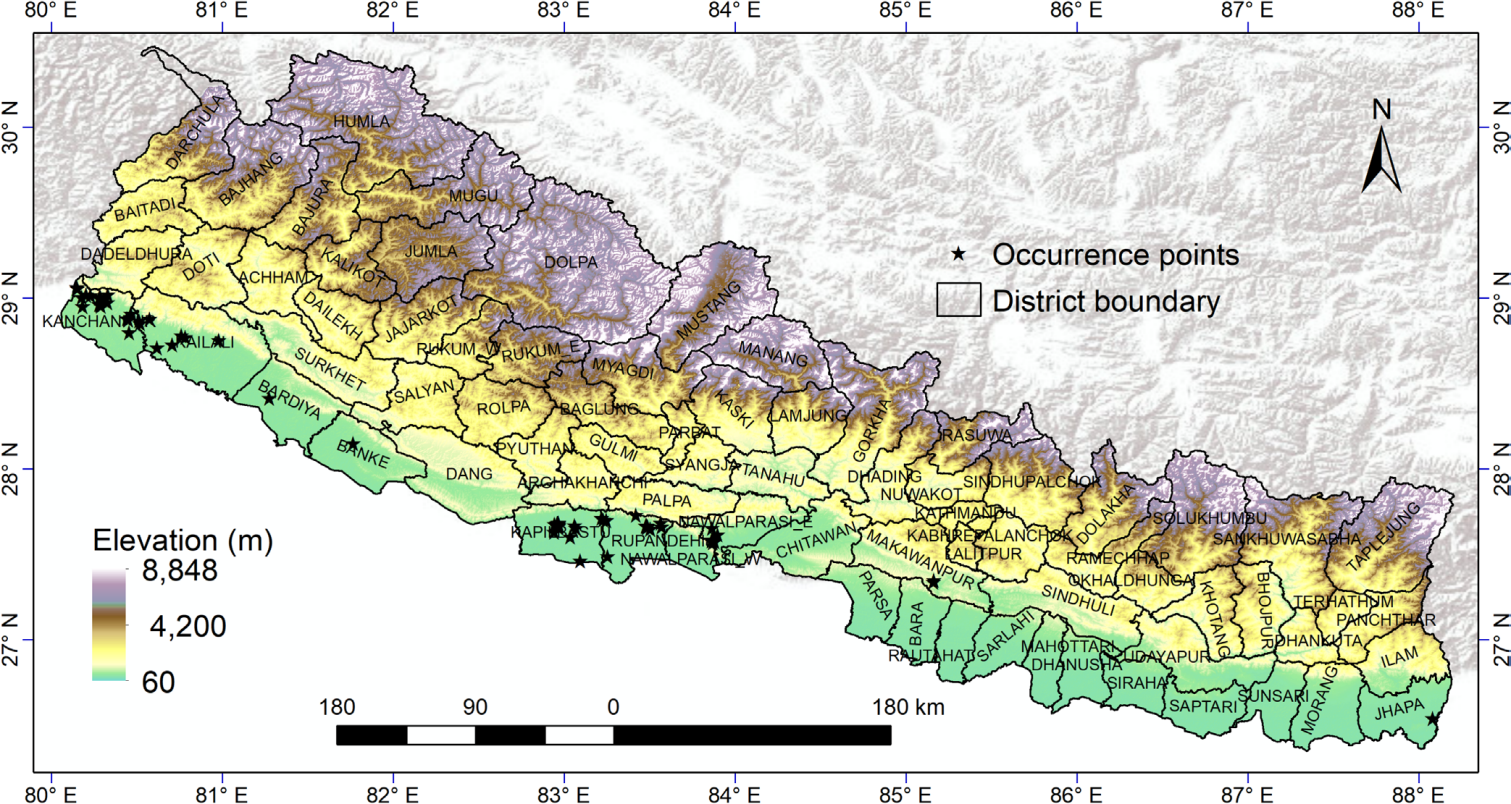
Study area map showing the distribution of occurrence points of *P. marsupium* in Nepal.

The study was conducted across Nepal, a region with diverse climatic zones ranging from the tropical Terai plains to the high elevation, providing a sharp environmental gradient for niche modeling. A total of 154 occurrence points were compiled from: field surveys (*n* = 89), herbarium specimens (KATH, TUCH, DoF; *n* = 37), and published literature plus online databases (GBIF; *n* = 28). After removing duplicates (*n* = 12) and erroneous records (*n* = 5), 137 points remained. Spatial thinning at 1 km resolution reduced this to 129 unique localities used for modeling (Figure 1).

### 2.2 Bioclimatic, Environmental, and Other Associated Variables

The 19 bioclimatic layers were downloaded from WorldClim 2.1 (https://www.worldclim.org/). Additionally, topographic variables (slope, aspect) were derived from GMTED2010 (https://earthexplorer.usgs.gov) (Table S1). The anthropogenic variable consisted of the cumulative human footprint index from the Global Human Footprint dataset (Mu et al. 2022). Edaphic variables (bulk density, total nitrogen content, soil organic carbon content) were downloaded from SoilGrids 2.0 (Poggio et al. 2021). The redundancy of 19 bioclimatic variables, with overlapping environmental gradients and intercorrelated values, may lead to unstable model estimation and poor ecological interpretation. To minimize multicollinearity and overfitting, variable selection was conducted using pairwise Pearson correlation coefficients (r). First, the 19 initial bioclimatic variables were evaluated, and for pairs with high collinearity (|r| > 0.7) (Figure S1), the variable with greater ecological relevance to the species' distribution was retained, reducing the set to six bioclimatic variables (Schymanski et al. 2013). After that, to ensure no overlapping gradients existed between different variable types, these six bioclimatic variables were pooled with the six environmental variables (human footprint, slope, aspect, soil bulk density, soil nitrogen, soil organic carbon) and evaluated simultaneously. All pairwise correlation coefficients among the final 12 variables were used for the ensemble modeling (Figure S2).

### 2.3 Species Distribution Modeling

To predict the current and future potential distribution of *Pterocarpus marsupium* in Nepal, we employed the biomod2 platform (version 3.5.1; Thuiller et al. 2009) in R 4.2.2 (R Core Team 2025), a comprehensive ensemble modeling framework for species distribution modeling. Model integrates multiple statistical and machine learning algorithms to quantify relationships between species occurrences and environmental variables, thereby predicting spatial distribution patterns (Thuiller et al. 2016; Zhao et al. 2025). Initially, we employed eleven individual modeling algorithms fixed within the biomod2 platform (Table S2). To accommodate algorithms requiring absence information, we generated 10 replicates of 1000 randomly selected non‐presence points outside a 1 km buffer around known occurrence records. The buffer was chosen based on the species' limited seed dispersal (Thomson et al. 2011) to reduce the likelihood of assigning pseudo‐absences within potentially occupied areas. These non‐presence points were weighted using a target‐group background approach to minimize sampling bias (Barbet‐Massin et al. 2012). Within the biomod2 framework, these points were treated as background locations for Maxent and as pseudo‐absences for algorithms requiring presence–absence data (e.g., Random Forest, GLM, GAM, and related methods). Models that achieve True Skill Statistic (TSS) > 0.8 and Area Under the Curve (AUC) > 0.9 across all runs were retained for ensemble building. Ensemble projections used both committee averaging (CA) and exponential moving weighted mean (EMWmean). This final weighted‐mean ensemble output was utilized to generate the response curves for environmental predictors detailed in the Supporting Information (Figure S3).

For future projections, the bioclimatic data we utilized represented two periods: 2050s: 2041–2060; and 2090s (2081–2100), under SSP1‐2.6, SSP2‐4.5, SSP3‐7.0, and SSP5‐8.5 greenhouse gas emission scenarios from the Coupled Model Intercomparison Project6 (CMIP6) version. For each period, the Beijing Climate Center Climate System Model (BCC‐CSM2‐MR) developed at the National Climate Center (Wu et al. 2019) was selected. This model has been demonstrated in comparative regional assessments to exhibit low systematic biases in reproducing monsoon precipitation dynamics and temperature gradients over the complex topographies of the Himalayas and Nepal, making it a highly reliable standalone choice for ecological forecasting in this region (Wu et al. 2019).

### 2.4 Data Processing and Visualization

Visualization of habitat change more naturally, the continuous suitability scores (0–1) generated by the ensemble model were reclassified in ArcGIS 10.8 into four categories using an equal‐interval approach: unsuitable (0–0.25), minimally suitable (0.26–0.50), moderately suitable (0.51–0.75), and highly suitable (0.76–1.0). The baseline threshold of 0.25 separating unsuitable from suitable habitat was explicitly selected (Thuiller et al. 2009) because it corresponds to the ensemble model's optimized threshold that maximizes the TSS, providing a foundation for classification. Biologically, the inclusion of the minimally suitable class (0.26–0.50) is necessary because these transitional habitats represent critical range edges and potential migration pathways that the species can utilize during climate‐driven range shifts, even if they exhibit lower initial density or regeneration rates. The total suitable area was thus defined as the cumulative sum of the minimal, moderate, and high‐suitability classes, and suitability maps were generated accordingly. Potential expansion pathways, geographic centroids of suitable habitats were calculated for each future period, and centroid shift trajectories were mapped in ArcGIS 10.8. Spatial dynamics change analysis was conducted in R (R Core Team 2025), comparing current and future suitability to estimate changes in loss, gain, and net changes of suitable areas.

### 2.5 Protected Area Gap Analysis

Spatial gap analysis was conducted for conservation efficacy of the existing protected area network for *Pterocarpus marsupium* under current and future climate scenarios. The shapefile of Nepal's Protected Areas (PAs) was obtained from the Department of National Parks and Wildlife Conservation (DNPWC). We calculated suitable habitat area inside and outside protected areas using the spatial analysis environment in ArcGIS 10.8, with a suitability threshold at 0.25. For each scenario, the total area (km^2^) of suitable habitat within the boundaries of protected areas (“Inside PAs”) and outside protected areas (“Outside PAs”) was calculated using pixel counts via the Zonal Histogram. These spatial metrics were then compiled to quantify the proportion (%) of protected versus vulnerable habitats across space and time.

## 3 Results

### 3.1 Model Evaluation

**FIGURE 2:**
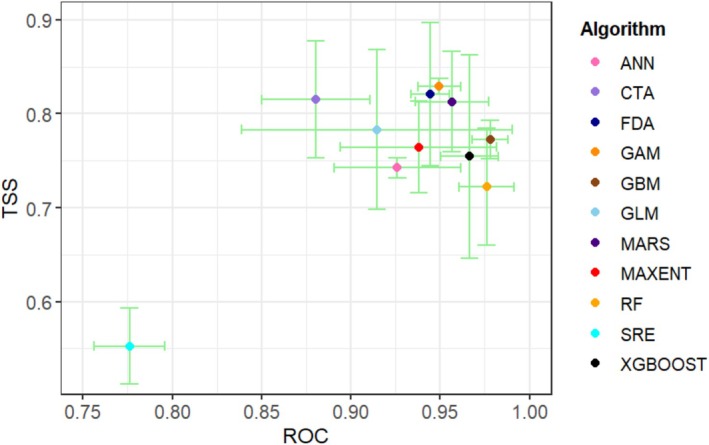
True skill statistics (TSS) and Area Under the Receiver Operating Characteristic Curve (AUC) values for each of the 11 individual algorithms.

**FIGURE 3:**
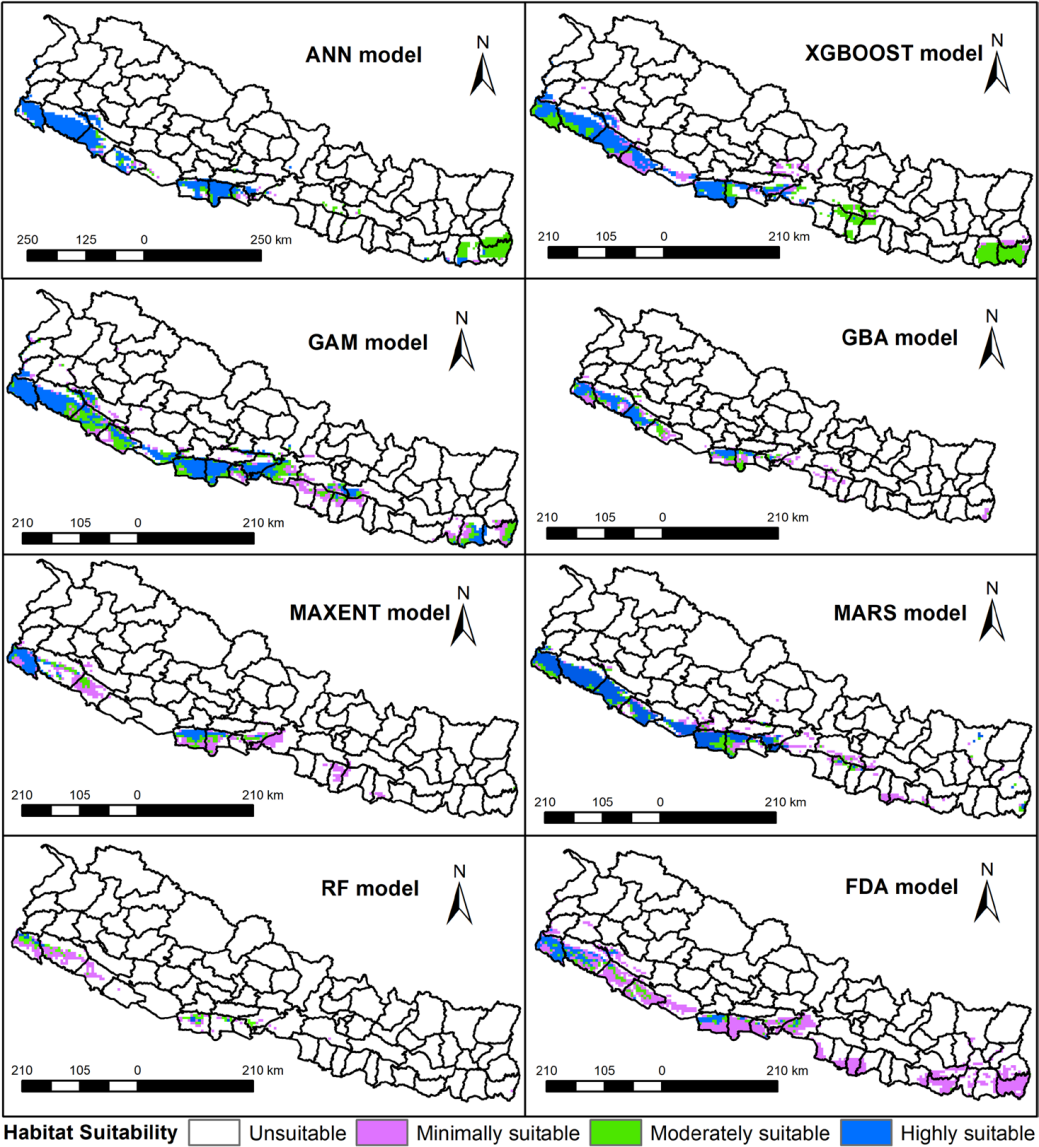
Current suitable habitat of *Pterocarpus marsupium* in Nepal determined by different algorithms.

The overall performance of the ensemble model was significant (AUC = 0.95, TSS = 0.89). The AUC and TSS values indicate that the final models are good for predicting *Pterocarpus marsupium* (Figure 2). Based on the evaluation metrics, Generalized Additive Model (GAM) emerged as the most robust individual algorithm, followed by Gradient Boosting Machine (GBM) and Multivariate Adaptive Regression Spline Model (MARS) (Table S3). Conversely, the Surface Range Envelop Model (SRE) recorded the lowest performance, indicating a more limited capacity to capture complex environmental relationships compared to more sophisticated algorithms. An ensemble modeling was implemented to minimize individual algorithmic bias and enhance spatial projection reliability, incorporating the top eight algorithms (Figure 3).

### 3.2 Ecological Determinants of Habitat Suitability

Thermal constraints and hydrological seasonality are the primary drivers of niche stability, with minimum temperature of the coldest month (Bio6) and precipitation seasonality (Bio15) emerging as the most significant predictors (0.092 and 0.091) (Table 1). The high contribution of Bio6, particularly within GAM (0.106) and MARS (0.097), points to a rigorous thermal bottleneck. Functional response curves reveal a critical physiological threshold: habitat suitability remains negligible below 0°C and rises precipitously between 5°C and 10°C, suggesting that winter frost persistence acts as the primary filter preventing poleward or upslope range expansion (Figure S3).

**TABLE 1:** Relative importance scores of environmental variables across the 11 individual algorithms used for *Pterocarpus marsupium* modeling in Nepal; calculated under current baseline environmental conditions within the biomod2 ensemble framework.

Bioclimatic variables	Individual species distribution algorithms											
	ANN	CTA	FDA	GAM	GBM	GLM	MARS	MAXENT	RF	SRE	XGBOOST	Mean
Bio1	0.083	0.079	0.080	0.084	0.082	0.101	0.085	0.085	0.084	0.091	0.080	0.084
Bio3	0.085	0.091	0.080	0.075	0.091	0.082	0.078	0.083	0.084	0.085	0.085	0.084
Bio6	0.089	0.103	0.087	0.106	0.092	0.095	0.097	0.091	0.085	0.090	0.095	0.092
Bio12	0.086	0.078	0.080	0.076	0.080	0.077	0.079	0.084	0.083	0.083	0.081	0.080
Bio14	0.085	0.078	0.080	0.074	0.080	0.082	0.079	0.081	0.083	0.083	0.080	0.080
Bio15	0.089	0.100	0.116	0.100	0.091	0.087	0.097	0.083	0.084	0.090	0.095	0.091
Human footprint	0.076	0.079	0.080	0.074	0.084	0.076	0.083	0.083	0.083	0.084	0.082	0.082
Slope	0.086	0.078	0.080	0.074	0.080	0.074	0.078	0.081	0.083	0.086	0.080	0.080
Aspect	0.075	0.078	0.080	0.074	0.080	0.073	0.078	0.080	0.083	0.074	0.080	0.078
Soil bulk density	0.074	0.078	0.080	0.085	0.080	0.078	0.078	0.082	0.083	0.084	0.080	0.080
Soil nitrogen	0.084	0.078	0.080	0.079	0.080	0.083	0.080	0.082	0.083	0.074	0.080	0.080
Soil organic carbon	0.088	0.078	0.080	0.100	0.080	0.093	0.087	0.083	0.083	0.075	0.082	0.083

Beyond bioclimatic variables, the model highlights the role of edaphic properties and anthropogenic pressure in enhancing the species' realized niche. Soil Organic Carbon (0.083) and Soil Bulk Density (0.080) showed high‐density, mineral‐rich substrates, reflecting the species' competitive advantage in specific successional forest stages. The Human Footprint index showed a non‐linear correlation, with peak suitability between values of 10–15. This suggests a hypothesis that moderately modified landscapes may offer suitable conditions, but causal mechanisms require field validation.

### 3.3 Current Potential Distribution of *Pterocarpus marsupium* in Nepal

**FIGURE 4:**
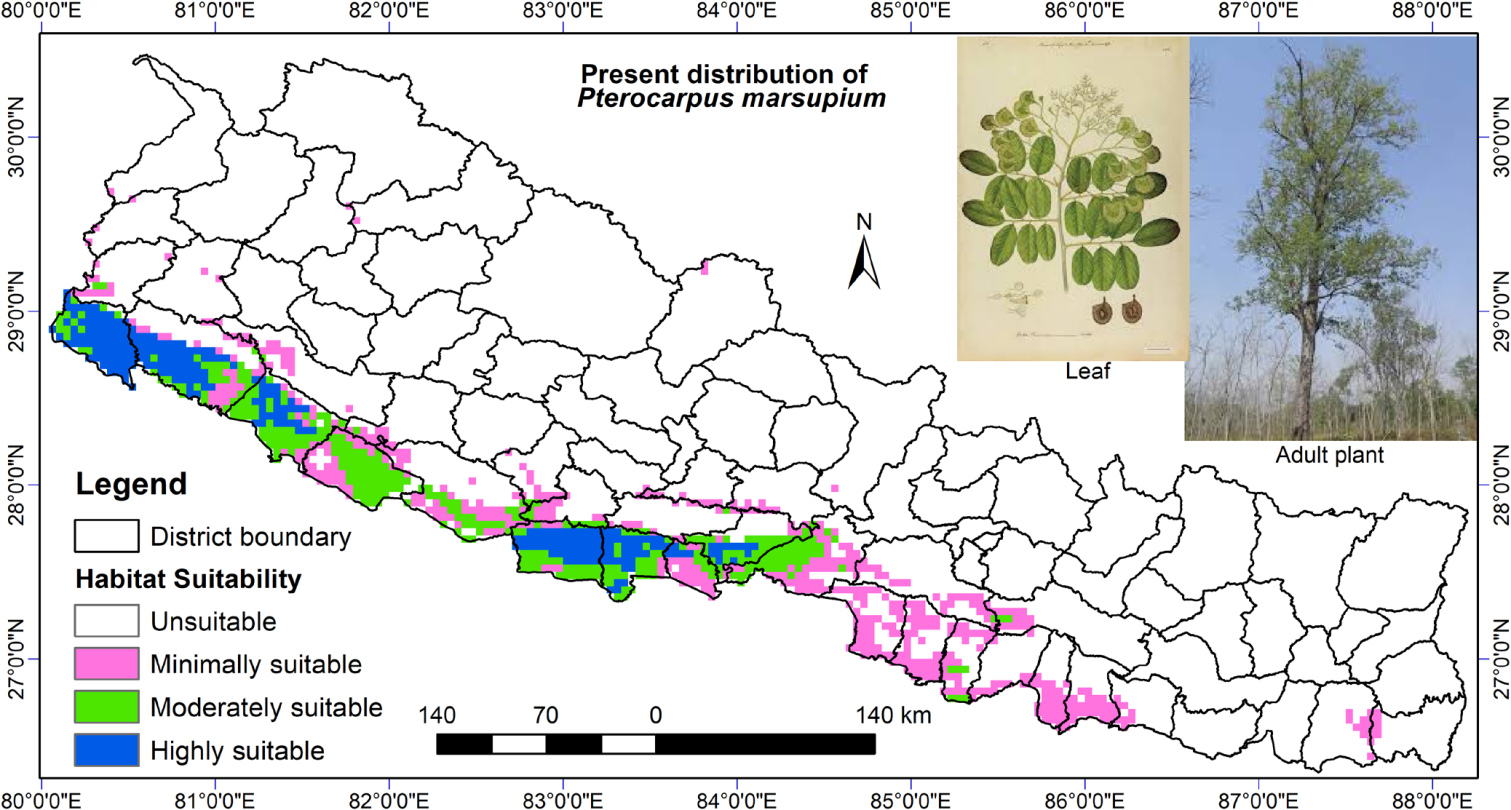
Ensemble‐modeled current potential distribution of *Pterocarpus marsupium* in Nepal.

Based on the ensemble model projections, the current potential distribution of *Pterocarpus marsupium* is primarily within the Terai and lower Siwalik regions, forming a narrow east–west band along the southern lowlands (Figure 4). Only limited, isolated patches of suitability were spotted outside this belt, suggesting strong climatic and edaphic constraints on the species' present distribution. Moderately suitable habitats appear as small, fragmented patches with irregular distributions, occurring irregularly in several regions, particularly in eastern Nepal. The total suitable habitat area under current climatic conditions is estimated at 11,154 km2, accounting for approximately 7.6% of Nepal's total land area.

### 3.4 Projected Future Distribution of *Pterocarpus marsupium* Under Climate Change Scenarios

The spatial projections for *P. marsupium* demonstrate a significant, non‐linear redistribution of suitable bioclimatic covers across Nepal through the mid‐ (2050s) and late‐century (2090s). Under all Shared Socioeconomic Pathways (SSPs), the model predicts a consistent northward expansion toward the mid‐hills, suggesting a thermic‐driven migration into higher elevation gradients as current low‐altitude habitats become increasingly suitable (Table 2). All scenarios produced gains in suitable habitat, although the magnitude and timing differed substantially. Under the low‐emission pathway (SSP1‐2.6), losses remained modest in the 2050s (14.6%) but escalated to 50.2% by the 2090s; nevertheless, extensive new areas became suitable, with potential climatically suitable areas of 163.5% and 40.6%, respectively. The intermediate pathway (SSP2‐4.5) produced the largest net expansions overall (312.7% in the 2050s and 185.8% in the 2090s), driven by exceptionally high gains (323.1% and 197.7%) against limited losses (< 12%). Under the high‐emission pathway (SSP3‐7.0), the 2050s showed the greatest proportional loss (54.7%), yet potential habitat increased by 58.9%; by the 2090s, losses declined, and gains surged, resulting in a 273.9% net expansion. Even under the very‐high‐emission pathway (SSP5‐8.5), the 2050s exhibited a net gain of 154.8%.

**TABLE 2:** Statistical analysis of the changes in the suitability of *Pterocarpus marsupium* in Nepal.

Scenario	Loss (km^2^)	Loss (%)	Stable (km^2^)	Stable (%)	Gain (km^2^)	Gain (%)	Net Change (%)
Present—SSP126 (2050s)	1629	14.6	9525	85.4	19,870	178.1	163.5
Present—SSP126 (2090s)	5599	50.2	5555	49.8	10,127	90.8	40.6
Present—SSP245 (2050s)	1154	10.3	10,000	89.7	36,037	323.1	312.7
Present—SSP245 (2090s)	1329	11.9	9825	88.1	22,053	197.7	185.8
Present—SSP370 (2050s)	6105	54.7	5049	45.3	12,675	113.6	58.9
Present—SSP370 (2090s)	2411	21.6	8743	78.4	32,962	295.5	273.9
Present—SSP585 (2050s)	1417	12.7	9737	87.3	18,680	167.5	154.8

**FIGURE 5:**
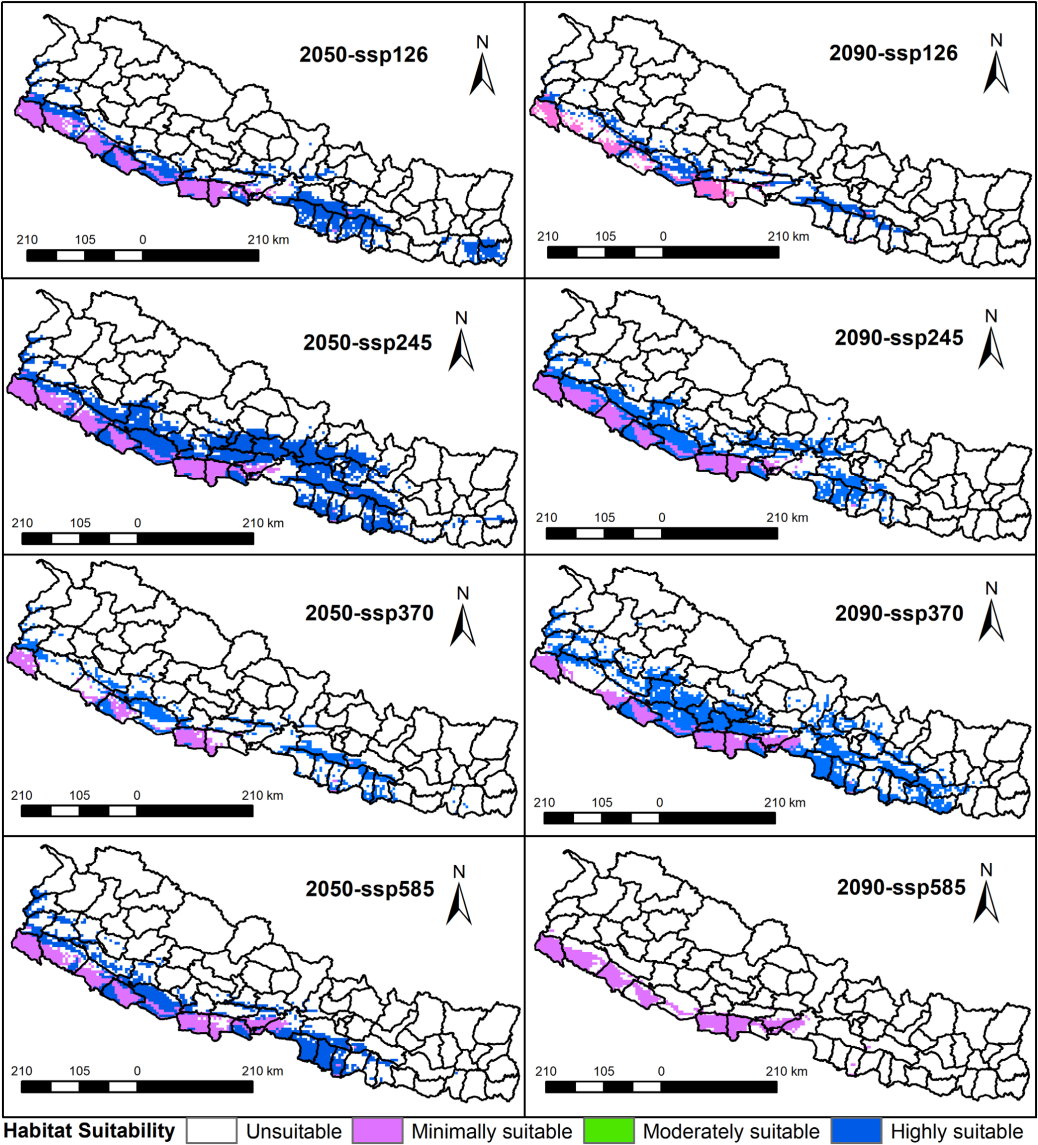
Ensemble‐predicted habitat suitability of *Pterocarpus marsupium* in Nepal under future climatic scenarios for the 2050s and 2090s under four SSP scenarios (SSP1‐2.6, SSP2‐4.5, SSP3‐7.0, SSP5‐8.5).

Spatially, suitable habitat (minimally to highly suitable) was consistently concentrated along the southern terai lowlands and Siwalik foothills, with scattered patches extending into the mid‐hills (Figure 5). Under SSP1‐2.6, a near‐continuous band of moderately to highly suitable habitat persisted along the southern border in both 2050 and 2090, with modest northward expansion in eastern and western districts. SSP2‐4.5 projections showed the most pronounced increase in highly suitable habitat, particularly in central‐southern and eastern districts, where dark‐green (highly suitable) zones expanded markedly by 2090. In contrast, SSP3‐7.0 produced more fragmented patterns in the 2050s, with larger unsuitable areas in the central Terai, followed by extensive consolidation of moderately and highly suitable habitat across eastern and western lowlands by 2090. Under SSP5‐8.5 (2050s), highly suitable patches were prominent at the western and eastern extremities; the 2090 map indicated a shift toward predominantly minimally suitable conditions across much of the south, accompanied by contraction of the darkest suitability classes.

### 3.5 Centroids Shifts of *Pterocarpus marsupium* Under Future Climate Change Conditions

**FIGURE 6:**
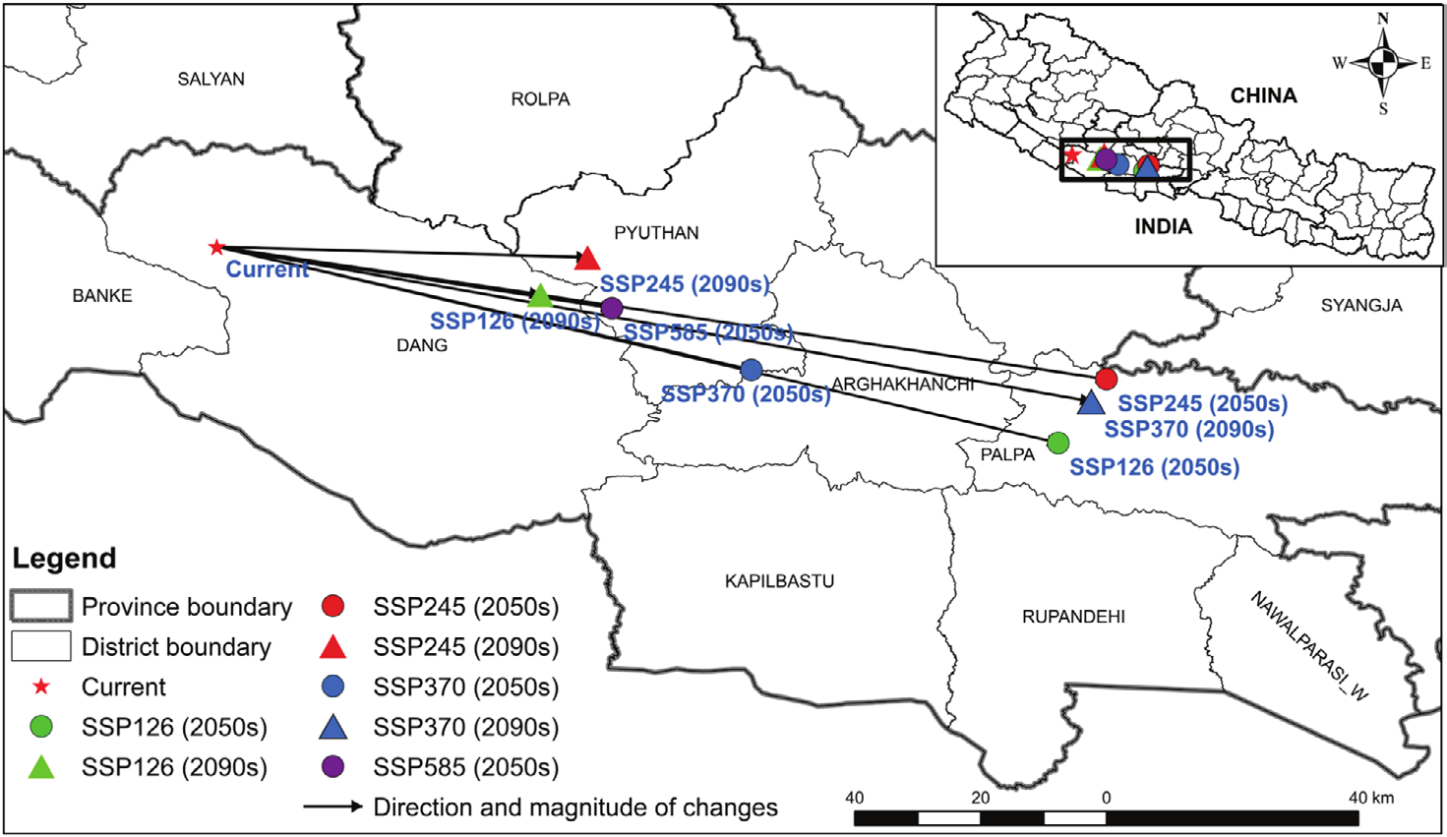
Geographic centroid migration pathways of predicted suitable habitats for *Pterocarpus marsupium* in Nepal under current and future (2050s and 2090s) climate scenarios. Arrows indicate the direction and magnitude of centroid shifts relative to the current baseline ecosystem (Dang district).

The centroid of the total suitable habitats of *P. marsupium* under various climatic scenarios is depicted in Figure 6. Under current climate conditions, the centroid of suitable habitats is located in the Dang district of Lumbini Province. All future scenarios for which data were available produced consistent southeastward shifts in centroid position. Displacement distances ranged from 46 km (SSP1‐2.62090s) to 127 km (SSP2‐4.52050s), with shift directions uniformly between 92° and 104°. The largest displacements occurred under SSP2‐4.52050s (127 km at 99°) and SSP3‐7.0 (2090s) (125 km at 101°), while the smallest shift was recorded under SSP1‐2.6 in 2090 (46 km at 99°). Every shift comprised a pronounced eastward component accompanied by a modest southward component, consistent with the expansion of suitable habitat into eastern districts of the terai and Siwalik foothills evident in the habitat‐suitability maps. This southeastward centroid shift indicates a horizontal geographic redistribution of the core suitable habitat within the lowlands, which occurs simultaneously with the distinct leading‐edge altitudinal expansion of the species into the northern mid‐hill regions.

### 3.6 Habitat Suitability of *Pterocarpus marsupium* in Protected Areas

**FIGURE 7:**
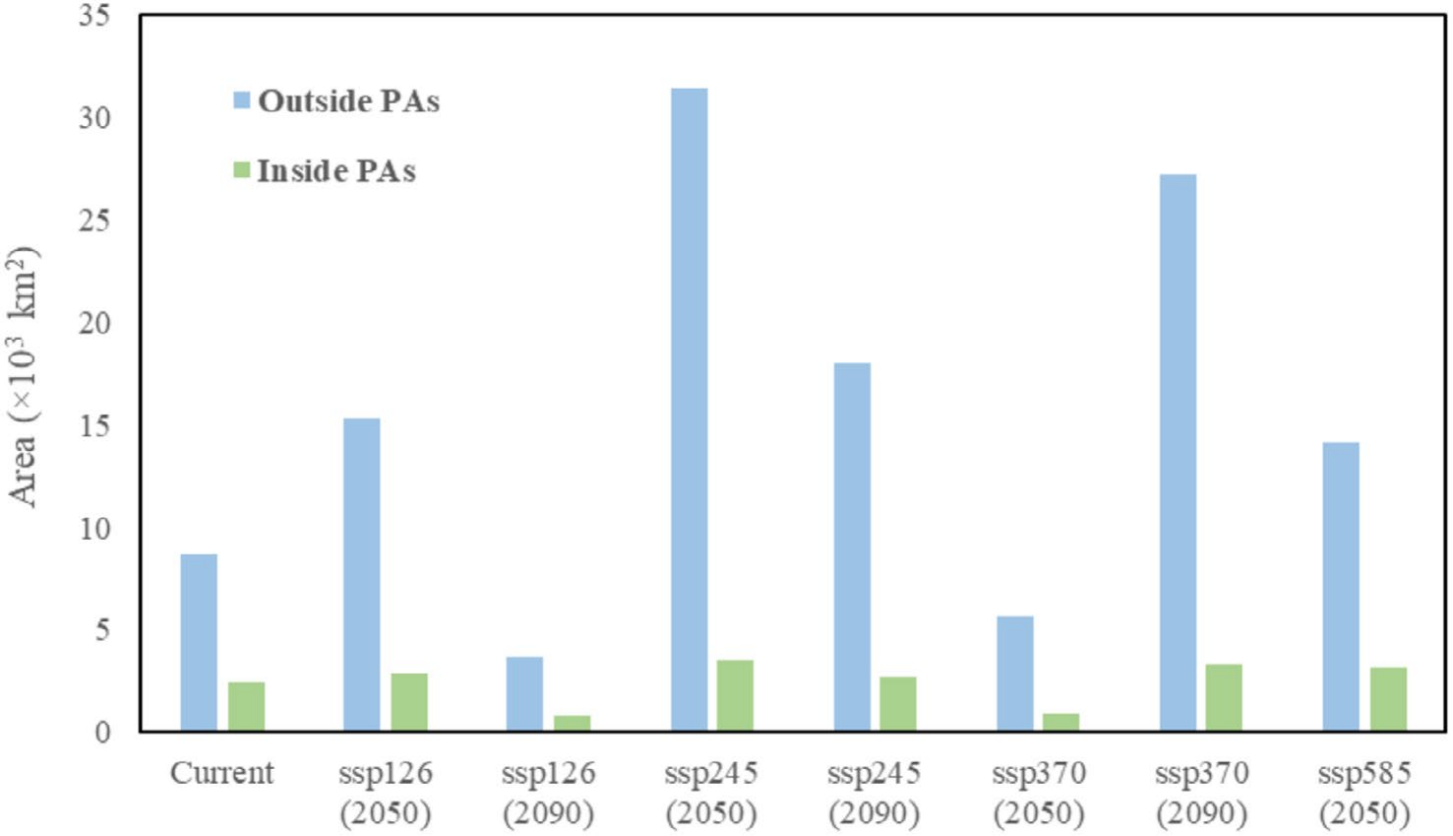
Predicted suitable habitat area (km^2^) for *Pterocarpus marsupium* inside and outside protected areas in Nepal.

The spatial overlay analysis shows a significantly suitable habitat of *P. marsupium* existing in the PAs. Under current climatic conditions, 2453.88 km^2^ (21.9%) of the total suitable habitat is in PAs, whereas 8700.12 km^2^ (78.1%) is distributed outside the PAs. Future projections indicate the conservation gap will persist through the 2050s and 2090s (Figure 7). In the 2050s, under low‐to‐moderate emission scenarios (SSP1‐2.6 and SSP2‐4.5), the suitable habitat expands, yet the area expands, and the proportion inside PAs remains low at 2917.8 km^2^ (15.9%) and 3487.8 km^2^ (9.8%), respectively.

## 4 Discussion

### 4.1 Ecological Niche and Environmental Determinants

The ensemble model shows the minimum temperature of the coldest month (Bio6) as the dominant predictor of *Pterocarpus marsupium* distribution in Nepal. It connects with a well‐established pattern for tropical tree species at their northern range margins: winter frost acts as a physiological filter that prevents upslope and poleward expansion (Cunningham and Read 2003; Feeley et al. 2020). The sharp decline in suitability below 5°C–10°C suggests a critical thermal threshold, below which either direct tissue damage or indirect effects on regeneration become prohibitive. Field surveys confirm that the species is virtually absent from areas with regular frost events (Bhattarai et al. 2021), validating our model's inference that cold intolerance, not growing‐season warmth, defines the current range limit. Precipitation seasonality (Bio15) contributed almost equally to model performance; it reflects the species' evolutionary adaptation to strongly seasonal tropical monsoon climates. *P. marsupium* leaves during the dry season and flowers with the onset of the monsoon (Singh and Kushwaha 2005), a phenological strategy that requires predictable inter‐annual rainfall contrasts. In addition, the contribution of dry‐month precipitation (Bio14) indicates that even within a seasonally dry regime, a minimum moisture baseline is necessary to avoid desiccation mortality, particularly for seedlings, which lack deep root systems (Poorter and Markesteijn 2008).

Edaphic variables, mainly soil organic carbon and bulk density, further refined the realized niche, with importance scores only slightly below those of the top bioclimatic predictors. Local‐scale soil heterogeneity has been shown to create micro‐refugia or dispersal barriers for many tropical tree species (John et al. 2007; Corlett 2016), and our results suggest that *P. marsupium* is no exception. The Human Footprint index shows that suitability peaks at intermediate scores of 10–15. Regionally, this range defines rural socio‐ecological zones characterized by low‐intensity agroforestry and traditional community forest use, rather than urban centers. While this pattern aligns with the Intermediate Disturbance Hypothesis, these broad spatial correlations should be interpreted with caution before assuming landscape‐level footprints directly translate to improved stand‐level seedling recruitment. This pattern aligns with the Intermediate Disturbance Hypothesis (Connell 1978) and has been observed for other disturbance‐adapted tree species in South Asian managed landscapes (Pandey et al. 2020; Sapkota et al. 2009). Moderate disturbance, such as selective harvesting, rotational grazing, or traditional agroforestry, may reduce competition from fast‐growing pioneers, expose mineral soil for seed germination, and maintain a semi‐open canopy that favors *P. marsupium* regeneration (Chhetri et al. 2019).

### 4.2 Current Distribution and Habitat Fragmentation

The current suitable habitat area confirms that *P. marsupium* is the most spatially restricted timber tree species in the country (GoN 2021), comparable in limited extent to *Dalbergia latifolia* in India (Rathore et al. 2019) and to other rare *Pterocarpus* species. The distribution is fragmented, with suitable patches concentrated in western and central Terai districts (Kailali, Kanchanpur, Dang, Banke, Bardiya, Chitwan) and only sparse, isolated suitability in the eastern Terai. This spatial pattern is consistent with field‐based assessments (Pant and Yadav 2013; Paneru et al. 2018) and reflects a combination of natural edaphic discontinuities and land‐use pressure. Eastern Nepal has a higher population density and a longer history of agricultural conversion, which may have extirpated many populations before they could be recorded (Gautam et al. 2019).

Low‐elevation areas in the Terai are predicted as unsuitable, despite being within the species' reported elevational range (100–640 m; DoF 2018). This indicates that elevation alone is a poor predictor. Instead, the interaction of temperature seasonality, dry‐month precipitation, and soil properties creates a complex suitability surface. Some lowland areas are too dry; others have unfavorable soil texture or organic carbon content; and yet others suffer from excessive human footprint.

### 4.3 Future Projections and Climate Refugia

Under all four SSP scenarios, the model projects net climatic suitability expansion of 40–313%. If realized, this would be driven by climate warming, relaxing cold temperature constraints. However, actual range expansion will be limited by dispersal, soil suitability, and land‐use change (Chen et al. 2011; Lenoir and Svenning 2015). The largest expansion under SSP2‐4.5 suggests that an intermediate warming pathway, one that raises minimum temperatures without severely disrupting monsoon precipitation, may create optimal conditions for range expansion. Although the southeastward centroid shift (46–127 km, directions 92°–104°) initially appears to contradict the narrative of northward expansion, these two trends represent distinct components of range dynamics occurring simultaneously. The northward shift represents a leading‐edge expansion into higher elevations and mid‐hill areas as warming current winter frost bottlenecks. Conversely, the southeastward shift is driven by the horizontal redistribution of the core suitable habitat, where eastern and southeastern lowlands (Terai) experience habitat gains, and parts of the western terai undergo suitability declines. Conservation efforts that are currently strongholds in the western Terai may miss future population centers in the east. Moreover, the centroid shift implies that the species' range is not simply expanding isotropically; it is reorganizing spatially in response to heterogeneous changes in precipitation seasonality and temperature.

The pronounced differences among SSP scenarios underscore deep uncertainty. SSP3‐7.0 shows a 54.7% loss by the 2050s, followed by a 274% net gain by the 2090s, a non‐linear path that would be extremely challenging for long‐lived tree species to track. Such non‐linearities arise from competing effects; initial precipitation regime shifts may contract habitat before thermal constraints are sufficiently relaxed to allow expansion (Urban 2015). For conservation planning, static reserve networks designed under current conditions are unlikely to be adequate (Hannah et al. 2007). Adaptive management frameworks that allow for dynamic reallocation of protection and restoration efforts will be necessary. Under the high emission scenario (SSP5‐8.5), the 2090s projection shows predominantly low‐suitability habitat across much of the south, despite moderate gains in the 2050s. This suggests that beyond a certain warming threshold, combined heat and drought stress may exceed the species' physiological tolerance, reversing earlier gains (Warren et al. 2018). Avoiding this outcome requires rapid mitigation of greenhouse gas emissions, a global policy challenge beyond the scope of local conservation. Multi‐GCM ensembles would be valuable in future work to better quantify climate model uncertainty.

### 4.4 Conservation Implications and Management Priorities

Our results suggest that suitable habitat exists outside PAs. Therefore, investigating conservation opportunities in community‐managed and private lands may be worthwhile, pending local ecological assessments. Protection within only protected areas is insufficient for the long‐term conservation of this high‐value deciduous tree. To bridge this conservation gap, management priorities should focus on integrating biodiversity conservation into community‐managed forests and private forestry initiatives. The human footprint index indicates that intermediate levels of disturbance correspond with high habitat suitability. However, the relationship is correlative and based on a macro‐ecological index; it does not directly demonstrate that active community forest management improves field‐level persistence, recruitment, or regeneration (Chazdon 2008; Shrestha et al. 2020). Therefore, moderately disturbed landscapes should be interpreted cautiously as potential target zones that deserve further local‐scale empirical investigation. Future field research is necessary to verify whether existing community forestry practices correspond directly to this modeled optimal disturbance range and actively promote population demographics. The western and central Terai districts (Kailali, Kanchanpur, Dang, Banke, Bardiya) emerge as irreplaceable climate refugia areas that remain suitable across all future scenarios. These should be the area's highest priority for in situ conservation, including protection of mature seed‐bearing trees, enhancement of natural regeneration, and prevention of land‐use conversion (Keppel et al. 2012). Community forests within these districts with documented *P. marsupium* populations should receive targeted technical support and funding for species‐specific management planning. The observed Human Footprint Index suggests that *P. marsupium* may benefit from intermediate levels of disturbance. This aligns conceptually with the Intermediate Disturbance Hypothesis, but long‐term field monitoring is required to confirm whether moderate human activity actually improves seedling recruitment.

The projected expansion into mid‐hill regions indicates potential climatic suitability rather than accurate future occupancy. Because our model omits future land‐use dynamics, biotic interactions, and dispersal limits, these expansion zones represent hypotheses that require strict verification before implementing any assisted colonization. If assisted colonization is considered, a phased, experimental approach would be necessary: (1) soil surveys to verify edaphic suitability at proposed planting sites; (2) small‐scale pilot plantations using locally sourced seeds (ideally from similar elevation bands to maintain adaptive genetic variation); (3) long‐term monitoring of survival, growth, and reproduction; and (4) if pilots succeed, gradual scaling up (Hällfors et al. 2014). No active translocation is recommended based solely on these model results. The genetic implications of upward range shifts are not trivial; lowland populations may possess different adaptive traits than those required for mid‐hill conditions, and mixing provenances without testing could lead to maladaptation (Prober et al. 2015; Breed et al. 2018).

The legal framework in Nepal already permits *P. marsupium* utilization from managed forests (*Government of Nepal* 1993, 2001). This creates policy space for benefit‐sharing mechanisms that channel timber revenues into regeneration activities, a model that has worked for other high‐value species in community forestry (Gilmour 2016). Developing science‐based harvesting guidelines that maintain population viability while providing economic incentives should be a near‐term priority (Newton 2008).

### 4.5 Study Limitation

The distribution model has limitations that should be considered when interpreting the projection results. First, the ensemble model's performance metrics were derived from random 80/20 train‐test splitting with spatial thinning of occurrence points to avoid pixel‐level sample duplication and spatial autocorrelation. Due to constrained geography, training and testing points may share similar environmental characteristics, potentially overestimating the model's ability to generalize to geographically distinct areas; spatial autocorrelation between training and testing sets likely inflated these values (Roberts et al. 2017). While our results provide a baseline for *P. marsupium* suitability in Nepal, future studies could employ more rigorous validation techniques to further assess model transferability across spatial scales. Similarly, overestimation has been documented for Himalayan species distribution models where occurrence records are spatially aggregated (Shrestha et al. 2021). Consequently, while our variable importance rankings and relative habitat suitability patterns are consistent, the absolute suitability values and the magnitude of projected range expansions are based on a single climate model (BCC‐CSM2‐MR). While this model performs well for the Himalayas, multi‐model ensembles typically provide more robust uncertainty quantification.

## 5 Conclusions

*Pterocarpus marsupium* occupies a narrow ecological niche defined by cold tolerance limits, monsoon seasonality, well‐drained fertile soils, and intermediate levels of anthropogenic disturbance. The current suitable habitat is restricted to 7.6% of Nepal, fragmented, and concentrated in the western and central Terai. While future climate warming is projected to expand potential habitat substantially under the selected GCM framework, actual occupancy will be restricted by natural dispersal limitations, future land‐use changes, and high‐elevation establishment constraints. The southeastward centroid shift indicates a spatial reorganization of suitable areas, requiring dynamic conservation planning. Conservation initiatives should investigate moderately disturbed landscapes and community forests as potential habitats alongside strict protected areas. Niche‐based assessments like this one can help generate hypotheses for proactive conservation planning, but they should be complemented with field studies on dispersal, regeneration, and land‐use change.

## Author Contributions

**Ripu Kunwar:** conceptualization (lead), data curation (lead), writing – original draft (lead). **Satyam Kumar Chaudhari:** formal analysis (lead), methodology (supporting), validation (equal), writing – original draft (equal). **Shreehari Bhattarai:** methodology (equal), writing – original draft (equal). **Binaya Adhikari:** validation (supporting), visualization (supporting). **Babita Khadka:** data curation (supporting), investigation (equal). **Gokarna J. Thapa:** methodology (supporting), writing – original draft (equal). **Anant Bhandari:** data curation (supporting), writing – original draft (equal). **Man Dev Bhatt:** data curation (supporting), writing – original draft (supporting).

## Funding

The authors have nothing to report.

## Conflicts of Interest

The authors declare no conflicts of interest.

## Supporting information


**Table S1:** List of Environmental variables used for the species distribution algorithm for *Pterocarpus marsupium* .
**Table S2:** List of Species distribution algorithms used in the biomod2 for *Pterocarpus marsupium* modeling.
**Table S3:** Evaluation metrics (AUC and TSS) of the ensemble model and individual species distribution algorithm for *Pterocarpus marsupium* .
**Figure S1:** Pearson correlation coefficient (r) matrix of the 19 bioclimatic variables.
**Figure S2:** Pearson correlation coefficient (r) matrix of the 12 final environmental predictor variables utilized in the ensemble species distribution modeling for *Pterocarpus marsupium* in Nepal.
**Figure S3:** Response curves for *Pterocarpus marsupium* generated from the ensemble weighted mean (EMWmean) using the biomod2 platform. Each curve shows the predicted habitat suitability (y‐axis, 0–1) as a function of a single environmental predictor, with all other predictors held at their median values.

## Data Availability

All the required data are uploaded as Supporting Information.
