# Supplementary material for: Predicting the Habitat Suitability for Endangered Tree Species Pterocarpus marsupium in Nepal, Using Ensemble Species Distribution Models
Source: Ecol Evol. 2026 Jul 10;16(7):e73990. doi: 10.1002/ece3.73990 (PMC13354544; doi:10.1002/ece3.73990)
Supplement: Supplementary file 1 — Table S1: List of Environmental variables used for the species distribution algorithm for Pterocarpus marsupium . Table S2: List of Species distribution algorithms used in the biomod2 for Pterocarpus marsupium modeling. Table S3: Evaluation metrics (AUC and TSS) of the ensemble model and individual species distribution algorithm for Pterocarpus marsupium . Figure S1: Pearson correlation coefficient (r) matrix of the 19 bioclimatic variables. Figure S2: Pearson correlation coefficient (r) matrix of the 12 final environmental predictor variables utilized in the ensemble species distribution modeling for Pterocarpus marsupium in Nepal. Figure S3: Response curves for Pterocarpus marsupium generated from the ensemble weighted mean (EMWmean) using the biomod2 platform. Each curve shows the predicted habitat suitability (y‐axis, 0–1) as a function of a single environmental predictor, with all other predictors held at their median values. [file ECE3-16-e73990-s001.docx]

**Supporting Documents**

**Predicting the habitat suitability for endangered tree species**

***Pterocarpus marsupium* in Nepal, using ensemble species distribution models**

Ripu Kunwar^1^**^†^**, Satyam Kumar Chaudhari^2,3^**^†^**^*^, Shreehari Bhattarai^4^, Binaya Adhikari^5^, Babita Khadka^6^, Gokarna J Thapa^7^, Anant Bhandari^7^, Man Dev Bhatt^6^

Supplementary File Information

Table S1 to S3

Figures S1 to S3

Table S1: List of Environmental variables used for the species distribution algorithm for *Pterocarpus marsupium*.

| Variable | Description | Abbreviation | Source |
| --- | --- | --- | --- |
| Bioclimatic | Annual mean temperature (°C) | BIO1 | WorldClim (Fick & Hijmans, 2017) |
|  | Isothermality (BIO2/BIO7 × 100) | BIO3 |  |
|  | Minimum temperature of coldest month (°C) | BIO6 |  |
|  | Annual precipitation (mm) | BIO12 |  |
|  | Precipitation of driest month (mm) | BIO14 |  |
|  | Precipitation seasonality (CV) | BIO15 |  |
| Anthropogenic | Composite index representing cumulative anthropogenic pressure (0–50), integrating built environments, croplands, population density, infrastructure, and accessibility. | Human footprint | Global Human Footprint (Mu et al., 2022) |
| Topographic | Slope angle (degrees) | SLOPE | GMTED2010 (Danielson et al., 2011) |
|  | Slope aspect (degrees) | ASPECT |  |
| Edaphic | Bulk density (g cm⁻³) | BD | Soil Grids (Poggio et al., 2021) |
|  | Total nitrogen content (g kg⁻¹) | SN |  |
|  | Soil organic carbon content (g kg⁻¹) | SOC |  |

Table S2: List of Species distribution algorithms used in the biomod2 for *Pterocarpus marsupium* modelling.

| Model Name | Model Code | References |
| --- | --- | --- |
| Generalized linear model | GLM | (Nelder et al., 1972) |
| Gradient boosting machine | GBM | (Friedman, 2001) |
| Generalized additive model | GAM | (Hastie, 2017) |
| Multivariate adaptive regression spline model | MARS | (Zakeri et al. 2010) |
| Classification tree analysis model | CTA | (Yarnold et al., 1997) |
| Artificial neural networks model | ANN | (Zupan, 1994) |
| Surface range envelop model | SRE | (Kruithof et al., 2006) |
| Flexible discriminant analysis model | FDA | (Hastie et al., 1994) |
| Random forest model | RF | (Rigatti, 2017) |
| Maximum entropy model | MaxEnt | (Phillips et al., 2006) |
| eXtreme Gradient Boosting | XGBOOST | (Chen & Guestrin, 2016) |

Table S3: Evaluation metrics (AUC and TSS) of the ensemble model and individual species distribution algorithm for *Pterocarpus marsupium*.

| Algorithm | Algorithm Code | Area Under the Curve (AUC) | True Skill Statistic (TSS) |
| --- | --- | --- | --- |
| Ensemble Model | EM | 0.95 | 0.89 |
| Generalized Additive Model | GAM | 0.99 | 0.943 |
| Gradient Boosting Machine | GBM | 0.96 | 0.92 |
| Multivariate Adaptive Regression Splines | MARS | 0.94 | 0.901 |
| eXtreme Gradient Boosting | XGBOOST | 0.936 | 0.923 |
| Random Forest | RF | 0.94 | 0.921 |
| Surface Range Envelope | SRE | 0.84 | 0.69 |
| Generalized Linear Model | GLM | 0.987 | 0.913 |
| Classification Tree Analysis | CTA | 0.967 | 0.91 |
| Artificial Neural Networks | ANN | 0.917 | 0.897 |
| Flexible Discriminant Analysis | FDA | 0.96 | 0.873 |
| Maximum Entropy | MaxEnt | 0.929 | 0.844 |


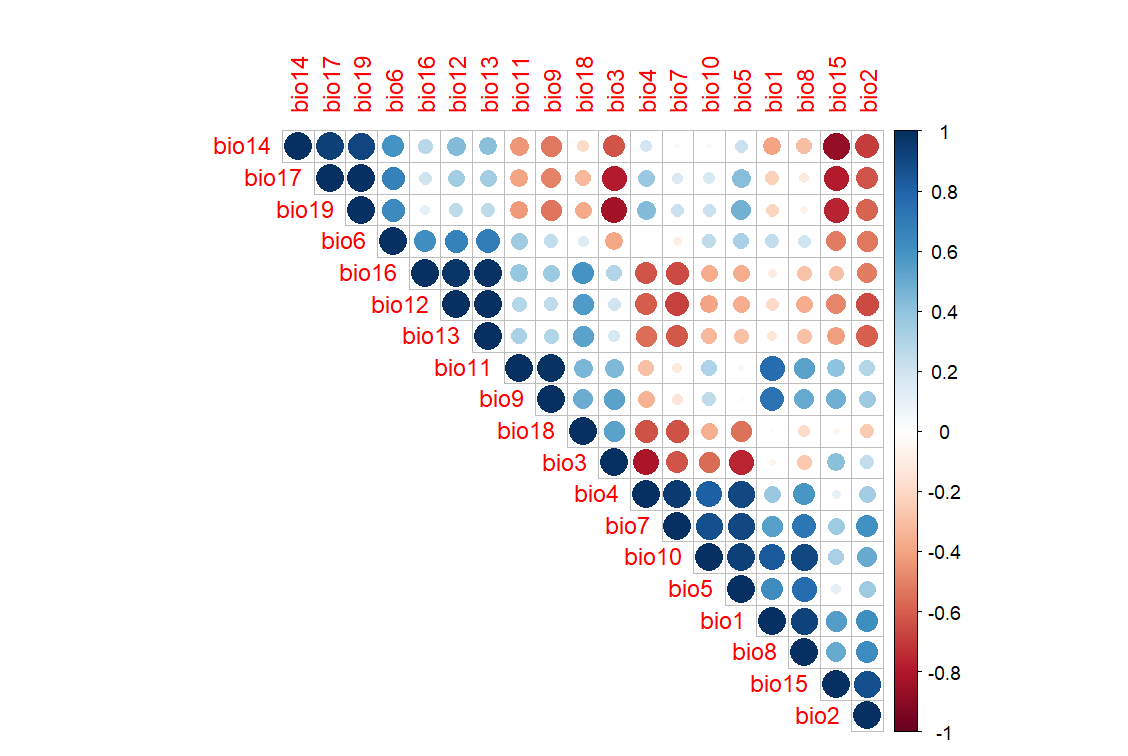


Figure S1: Pearson correlation coefficient (r) matrix of the 19 bioclimatic variables.


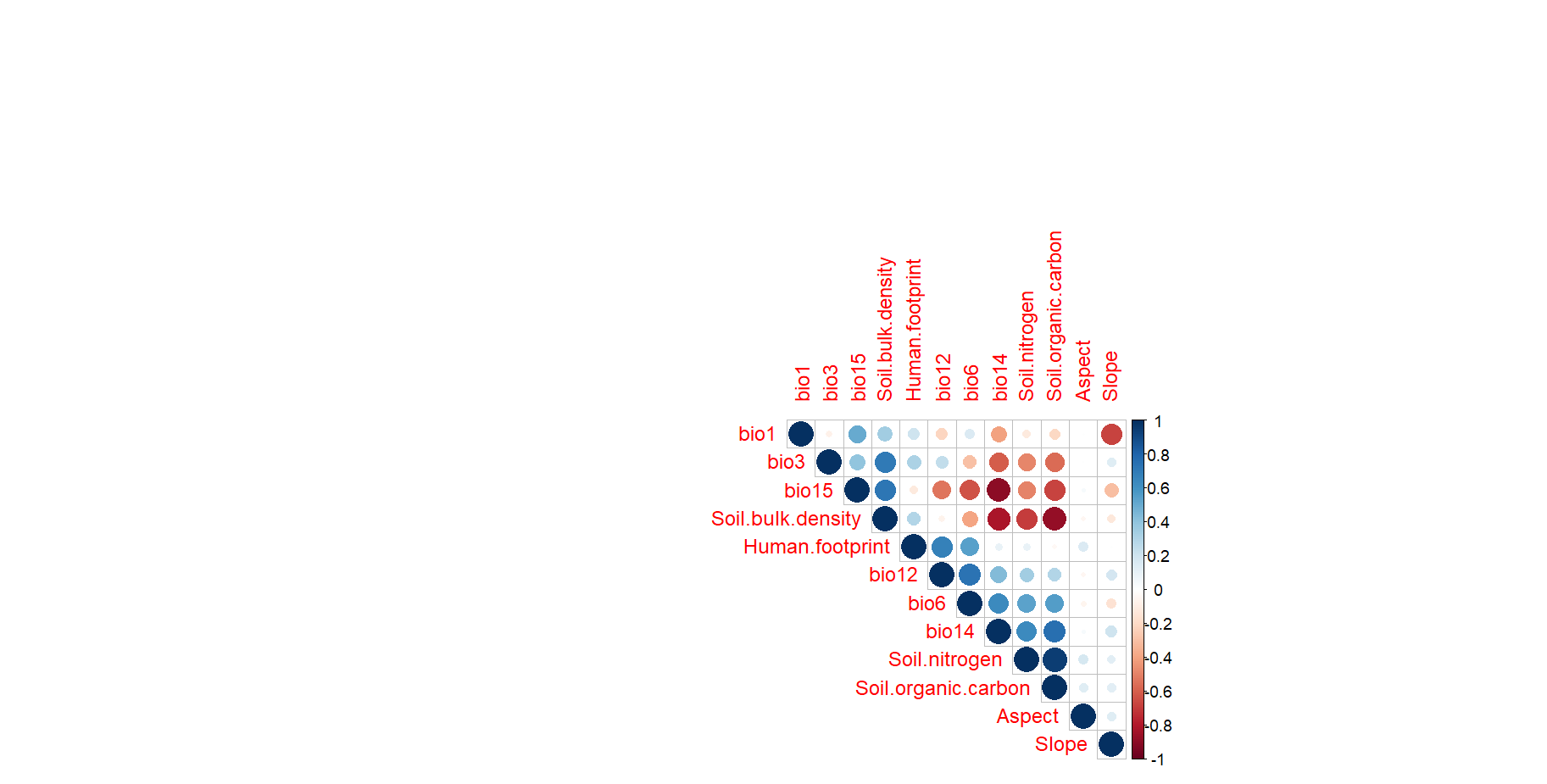


Figure S2: Pearson correlation coefficient (r) matrix of the 12 final environmental predictor variables utilized in the ensemble species distribution modeling for *Pterocarpus marsupium* in Nepal.


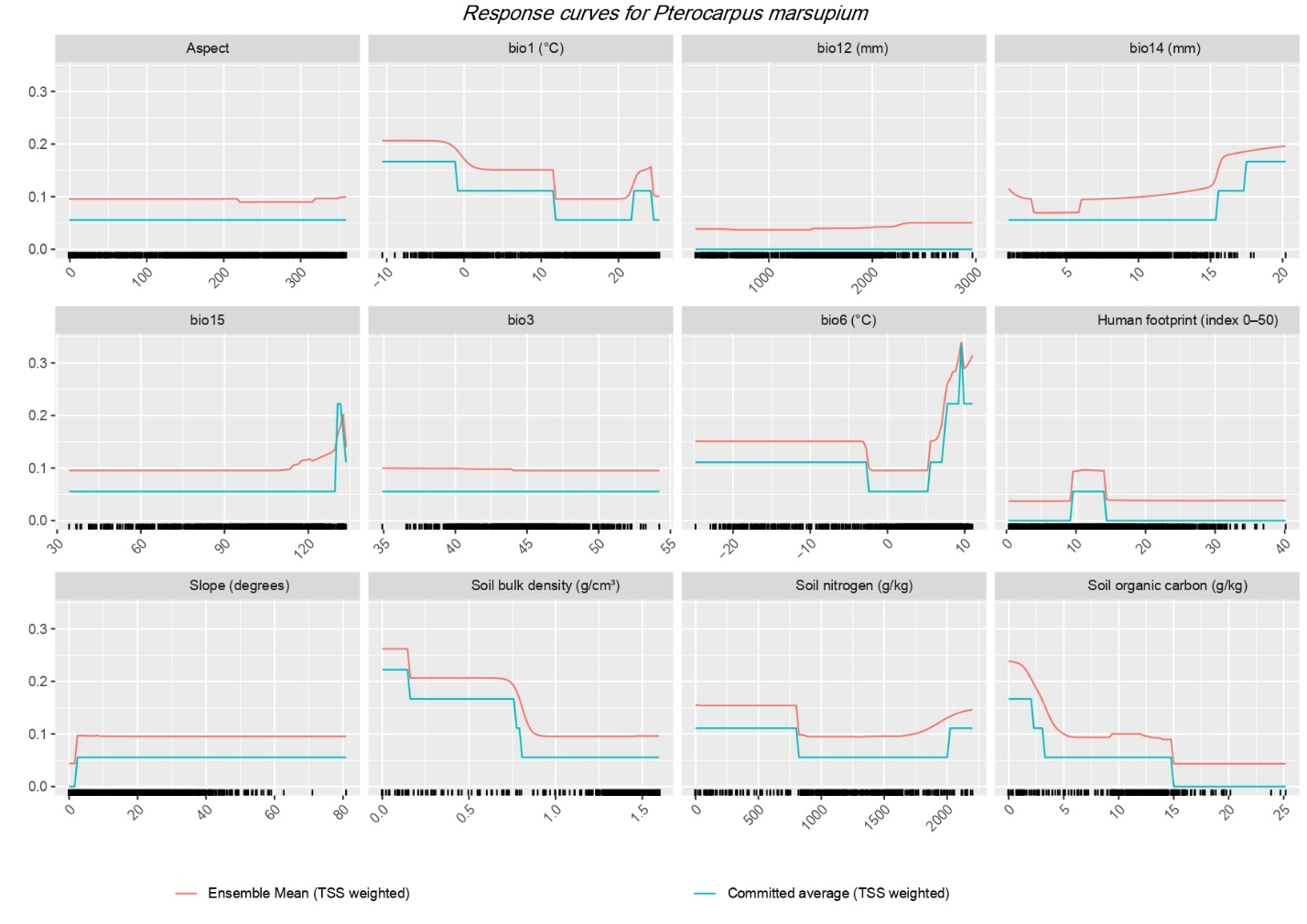


Figure S3: Response curves for *Pterocarpus marsupium* generated from the ensemble weighted mean (EMWmean) using the biomod2 platform. Each curve shows the predicted habitat suitability (y-axis, 0–1) as a function of a single environmental predictor, with all other predictors held at their median values.

***References:***

Chen, T., and Guestrin, C. 2016. XGBoost: A scalable tree boosting system. In Proceedings of the 22nd ACM SIGKDD International Conference on Knowledge Discovery and Data Mining (pp. 785–794). <https://doi.org/10.1145/2939672.2939785>

Danielson, J. J., and Gesch, D. B. 2011. *Global multi-resolution terrain elevation data 2010 (GMTED2010)* (U.S. Geological Survey Open-File Report 2011–1073). U.S. Geological Survey. [https://doi.org/10.3133/ofr20111073](https://www.google.com/search?q=https://doi.org/10.3133/ofr20111073)

Fick, S. E., and Hijmans, R. J. 2017. WorldClim 2: New 1‐km spatial resolution climate surfaces for global land areas. *International Journal of Climatology*, 37(12), 4302–4315. <https://doi.org/10.1002/joc.5086>

Friedman, J. H. 2001. Greedy function approximation: A gradient boosting machine. The Annals of Statistics, 29(5), 1189–1232. <https://doi.org/10.1214/aos/1013203451>

Hastie, T. 2017. Generalized Additive Models. Routledge. <https://doi.org/10.1201/9780203753781>

Hastie, T., Tibshirani, R., and Buja, A. 1994. Flexible discriminant analysis by optimal scoring. Journal of the American Statistical Association, 89(428), 1255–1270. <https://doi.org/10.1080/01621459.1994.10476866>

Kruithof, L., Thuiller, W., Antezana, M., & Schmatz, D. 2006. Surface range envelope model. BIOMOD User’s Manual v1. 0.

Mu, H., Li, X., Wen, Y., Huang, J., Du, P., Su, W., Miao, S., and Geng, M. 2022. A global record of annual terrestrial Human Footprint dataset from 2000 to 2018. *Scientific Data* 9(1): 176. <https://doi.org/10.1038/s41597-022-01284-8>

Nelder, J. A., and Wedderburn, R. W. M. 1972. Generalized linear models. Journal of the Royal Statistical Society: Series A (General), 135(3), 370–384. <https://doi.org/10.2304/2344614>

Phillips, S. J., Anderson, R. P., and Schapire, R. E. 2006. Maximum entropy modeling of species geographic distributions. Ecological Modelling, 190(3-4), 231–259. <https://doi.org/10.1016/j.ecolmodel.2005.03.026>

Poggio, L., de Sousa, L. M., Batjes, N. H., Heuvelink, G. B., Kempen, B., Ribeiro, E., and Rossiter, D. 2021. SoilGrids 2.0: Producing soil information for the globe with quantified spatial uncertainty. *Soil*, 7(1), 217–240. <https://doi.org/10.5194/soil-7-217-2021>

Rigatti, S. J. 2017. Random forest. Journal of Insurance Medicine, 47(1), 31–39. <https://doi.org/10.17849/insm-47-01-31-39.1>

Yarnold, P. R., Soltysik, R. C., and Bennett, C. L. 1997. Classification tree analysis. Statistics in Medicine, 16(13), 1451–1463.

Zakeri, I. F., Adolph, A. L., Puyau, M. R., Vohr, F. A., and Butte, N. F. 2010. Multivariate adaptive regression spline models for the prediction of energy expenditure in children and adolescents. Journal of Applied Physiology, 108(1), 128–136. <https://doi.org/10.1152/japplphysiol.00879.2009>

Zupan, J. 1994. Introduction to artificial neural network (ANN) methods: What they are and how they run. Acta Chimica Slovenica, 41(3), 327–342.
